# Interaction patterns and assembly mechanisms of dinoflagellates and diatoms in a coastal bay suffering from long-term eutrophication

**DOI:** 10.1128/msphere.00366-24

**Published:** 2024-06-28

**Authors:** Wenfei Miao, Shuqi Wang, Tenghui Lin, Yi Yan, Zhen Bao, Demin Zhang, Zhibing Jiang, Huajun Zhang

**Affiliations:** 1 School of Marine Sciences, Ningbo University, Ningbo, China; 2 State Key Laboratory for Managing Biotic and Chemical Threats to the Quality and Safety of Agro-products, Institute of Plant Virology, Ningbo University, Ningbo, China; 3 Key Laboratory of Applied Marine Biotechnology of Department of Education, Ningbo University, Ningbo, China; 4 State Key Laboratory of Satellite Ocean Environment Dynamics, Second Institute of Oceanography, Ministry of Natural Resources, Hangzhou, China; University of Wisconsin-Madison, Madison, Wisconsin, USA

**Keywords:** dinoflagellates, diatoms, assembly processes, interactions, stochasticity, eutrophication

## Abstract

Dinoflagellates and diatoms are highly prevalent and ecologically important phytoplankton in coastal waters, greatly contributing to primary productivity in marine ecosystems. Although their composition and diversity have been extensively elucidated in the open ocean, their interaction patterns and community assembly in long-term eutrophic coastal waters remain poorly understood. This investigation aimed to elucidate the seasonal successional patterns of dinoflagellates and diatoms by 18S rRNA gene amplicon sequencing in a semi-enclosed bay. The results revealed that dinoflagellate and diatom communities have pronounced seasonal succession patterns, which are primarily associated with temperature. Furthermore, the most prevalent species throughout the year were *Heterocapsa rotundata* and *Skeletonema costatum*. Moreover, the assembly of dinoflagellate and diatom communities was mainly dominated by stochastic processes, with drift being the major factor. The co-occurrence of dinoflagellates and diatoms showed seasonal patterns, with the highest interactions observed in autumn. In addition, interactions of Syndiniales with dinoflagellates and diatoms highlighted the roles of parasites in eutrophic conditions. *Flavobacteriaceae* and *Rhodobacteraceae* are the bacterial taxa that most frequently interacted with dinoflagellates and diatoms, with interactions between dinoflagellates and bacteria being more complex than those between diatoms and bacteria. Overall, this study provides results that deepen our understanding of the phytoplankton dynamics in coastal eutrophic waters.

**IMPORTANCE:**

Dinoflagellates and diatoms are major phytoplankton groups in coastal waters. The composition and diversity of dinoflagellates and diatoms in the open ocean have been well documented; however, it remains uncertain to what extent their adaptation to long-term eutrophic conditions influences their response to environmental disturbances. Here, we investigated the interactions and assembly processes of dinoflagellates and diatoms in a eutrophic bay throughout the whole year. Our findings revealed that interactions between dinoflagellates and diatoms are primarily shaped by seasonal transitions, while prolonged eutrophic conditions tend to amplify stochastic processes in community assembly. These findings provide novel perspectives on the influence of long-term eutrophication on phytoplankton dynamics within eutrophic waters.

## INTRODUCTION

Marine phytoplankton, comprising a diverse array of primary producers in the marine ecosystem, collectively contribute to approximately half of the global net primary productivity (1). Their relative abundance and realized niches can significantly impact global biogeochemical cycles, such as microbial loop and biological pump (2). Examining the underlying mechanisms that mediate phytoplankton community structure and drive current changes is therefore essential. The phytoplankton diversity and distribution have been extensively researched globally, with ecological processes such as environmental selection being the fundamental mechanism regulating their community structure (3, 4). For instance, temperature predominantly influences the distribution patterns of marine phytoplankton, directly by increasing metabolic rates and speciation and indirectly by altering circulation, trophic interactions, and stratification (4–6). Furthermore, light and nutrients, such as nitrogen, phosphorus, and silicate, have also been found as essential factors for the ecological distribution of phytoplankton (7). Due to the sensitivity of phytoplankton to environmental perturbations, investigating the mechanisms of their community assembly has become crucial and urgent.

Elucidating how ecological processes drive temporal patterns in community composition is an important topic of microbial ecology (8). Generally, ecological processes contained four main processes: selection, drift, speciation, and dispersal (9). Selection is a deterministic process and comprises heterogeneous and homogeneous selection (10). Drift occurs because of random fluctuations in microbial abundance, which might be due to processes like immigration, emigration, birth, and death. This stochastic phenomenon can lead to local extinction when microbial abundance is low (10). Speciation is the evolution of new species, whereas dispersal refers to the movement of microbes across space (9). To date, studies on the ecological processes that modulate marine microbial community compositions are primarily focused on prokaryotes and protists (3, 11–13), leaving the mechanisms influencing phytoplankton communities, particularly certain critical taxa, unclear. Moreover, most previous literature was conducted in oligotrophic environments or focused on the transition between oligotrophic and eutrophic conditions (14–16). In long-term eutrophic environments, phytoplankton may adapt to high-nutrient conditions, leading to completely distinct assembly mechanisms. In this case, the assembly processes modulating the assembly of phytoplankton communities need further investigation.

Xiangshan Bay (XSB) is a semi-enclosed subtropical bay situated in the northern East China Sea, characterized by a prolonged water residence time in its inner region (~80 days for 90% water exchanges). Since the 1980s, XSB has experienced significant anthropogenic disturbances, such as excessive mariculture activities and sewage discharge from industries and agriculture, which have degraded water quality and led to severe year-round eutrophication (17, 18). Additionally, an elevation in surface seawater temperature in the inner bay was observed due to thermal discharge from two thermal power plants (19), thereby further stimulating phytoplankton growth and the occurrence of frequent harmful algal blooms, particularly in cold seasons (19, 20). It has been reported that phytoplankton communities could change from diatoms to dinoflagellates in warmer waters due to thermal discharge (21). The succession of these phytoplankton species may significantly impact ecosystem function, consequently affecting coastal aquaculture and fisheries. Therefore, it is crucial and urgent to elucidate the assembly mechanisms of the phytoplankton communities in this highly fluctuating and eutrophic bay.

Dinoflagellates and diatoms are the two most abundant, diverse, and ecologically critical phytoplankton groups in the XSB (22). Most dinoﬂagellates and diatoms are large nanoplankton (10–20 µm) or microplankton (20–200 µm) and therefore can be precisely identified and quantified using traditional microscopy. It has been observed that alterations in environmental conditions, including nutrient concentration and structure, can effectively influence the successions of dinoflagellates and diatoms as they compete for resources and ecological niches based on their specialized adaptive strategies (23). Furthermore, their diverse intrinsic traits, including cell size and growth strategy, may influence the ecological processes that modulate community assembly (24). Understanding the seasonal abundance and assembly mechanisms of these phytoplankton in eutrophic waters can provide valuable insights into their succession mechanisms under constant high-nutrient conditions. Therefore, this study comprehensively analyzed the seasonal succession patterns and assembly mechanisms of dinoﬂagellates and diatoms in XSB. Monthly samples of microeukaryotes were collected throughout the year 2018 to investigate: (i) what factors control the seasonal successions of dinoﬂagellates and diatoms, (ii) whether their community assembly is shaped by similar or distinct ecological processes, and (iii) whether the interactions between them vary in different seasons. To date, this is the largest investigation to elucidate the seasonal patterns of phytoplankton communities in the XSB, which may provide valuable insights into the mechanisms behind harmful algal blooms induced by dinoﬂagellates and diatoms.

## MATERIALS AND METHODS

### Sample collection

A total of 156 surface seawater samples were collected monthly from 13 different sites in XSB from January to December 2018, with consistent intervals between samplings. Detailed sampling procedures can be found in our previous study (25). Briefly, from each site, 5 L of seawater was collected in a plastic bottle. For DNA extraction, water samples (700 mL) were first filtered through a 200 µm mesh, followed by further filtration through a 0.2 µm polycarbonate filter membrane (47 mm, Millipore, USA) to collect microbes and stored at −80°C until DNA extraction. The temperature, pH, and salinity of water were directly detected on board. Furthermore, standard methods were employed for the detection of dissolved oxygen, chemical oxygen demand (COD), Chl a content, silicate, phosphate, nitrite, ammonium, and nitrate (26). The detailed methods are reported in our previous study (25). To evaluate the levels of eutrophication, the nutritional quality index (NQI) was calculated based on inorganic nitrogen, Chl a, phosphate, and COD levels (27, 28).

Total DNA was extracted using a Power Soil DNA Isolation Kit (MO BIO, Carlsbad, CA). The previously reported protocol of PCR, purification, and library construction was followed (29). Finally, the V4 hypervariable region of the 18S rRNA gene was used for paired-end (2 × 300 bp) sequencing on an Illumina MiSeq platform.

### Data processing

Raw reads were processed in QIIME2 using DADA2 procedures (30, 31) to generate amplicon sequence variants (ASVs). Briefly, after primer trimming, DADA2 was employed for sequence denoising, which comprised the following steps: truncation of the forward (240 bp) and reverse reads (219 bp), quality filtering, merging of paired-end reads, chimeras removal, and ASVs generation. Taxonomic assignment of each ASV was conducted using the BLAST algorithm within QIIME2 against the Protistan Ribosomal Reference (PR2, v4.13) database (32). Furthermore, ASVs classified as chloroplasts, mitochondria, metazoan, and bacteria were discarded, and an ASV table containing 4,792 ASVs was acquired. Then, the frequency was normalized to 10,114 reads per sample, which was the lowest sequencing depth. ASVs were filtered to include only dinoflagellates and diatoms, resulting in 1,203 rarefied ASVs for dinoflagellates and 571 for diatoms. Sequence data are publicly available at the NCBI sequence read archive with BioProject ID PRJNA755531.

### Statistical analysis

All analyses were performed using R (3.3.3). Temporal changes in the community compositions at the phylum level were visualized across the whole year. The most abundant top 50 ASVs (top50) of both dinoﬂagellates and diatoms were selected and visualized using “pheatmap” package (33). Meanwhile, the sequences of these ASVs were selected to build a phylogenetic tree according to the Neighbor-Joining method using MEGA (34). The heatmap and phylogenetic tree were then combined. In addition, the correlations of relative abundance of dinoﬂagellates and diatoms with environmental factors were computed with Spearman’s rank correlation using the “psych” package (35). Furthermore, the relationships between the relative abundances of dinoflagellates and diatoms with temperature and NQI were also evaluated using the “lm” function in the “ggplot2” package (36). The Levins’ niche breadth of the dinoﬂagellates and diatoms was assessed using the “EcolUtils” package (37). The seasonality of the top50 ASVs of both phytoplankton was computed using the “seasonality.test” function with 1,000 permutations in the “EcolUtils” package (37). The relationship between seasonality and niche breadth of each ASV was fitted using the “lm” function in the “ggplot2” package (36). Furthermore, Spearman’s rank correlation was performed to evaluate the site occupancy of the ASVs, assessing the abundance-occupancy relationship between the number of sites they occupied and the log-transformed mean relative abundance of dinoﬂagellates and diatoms (38).

Differences in dinoﬂagellate and diatom communities were analyzed by non-metric multidimensional scaling (NMDS) based on Bray-Curtis dissimilarity in the “vegan” package (39). Moreover, the correlation between environmental factors (Euclidean distance) and variations of dinoﬂagellate and diatom communities (Bray-Curtis dissimilarity) was determined using the mantel tests with 999 permutations (40). Additionally, how environmental and geographic factors affect the variations of dinoﬂagellate and diatom communities were also identified by variation partitioning analysis (VPA) (39). Before the VPA, principal coordinates of neighbor matrices (PCNM) analysis of geographic factors were conducted. The acquired data were used for creating a matrix of geographic factors based on the latitude and longitude coordinates of sampling sites in the “vegan” package (39). Subsequently, a forward selection of environmental and geographic factors was carried out based on the significant explanation of factors to community variations (*P* < 0.05). Finally, the VPA was carried out using the selected environmental factors, PCNM variables, and geographic factors.

### Quantification of community assembly processes

Based on the framework proposed by Stegen et al. (41), null model analysis was performed to assess the β-nearest taxon index (βNTI) for clarifying roles of environmental selection (|βNTI| > 2) and stochastic processes (|βNTI| < 2). To infer the sub-ecological processes within stochastic processes, the Raup-Crick (RC_bray_) index was calculated to assess the roles of drift, dispersal limitation, and homogenizing dispersal (10, 41). These methods are reported in detail in our previous study (25). When community assembly is extremely stochastic, the average βNTI values may closely approximate the average null expectation in null model analysis; in this scenario, the accurate value of stochasticity (i.e., 100%) can be calculated by stochastic ratio (ST). The analysis of ST was determined using the tNST function in the “NST” package in R (8).

### Co-occurrence analysis of the microbial interactions

Co-occurrence networks were constructed to estimate species interaction patterns. For interactions between dinoflagellates and diatoms, only ASVs with a frequency of more than five were retained for co-occurrence analysis. Correlation networks were constructed based on Spearman’s rank correlation coefficients (|ρ| > 0.6 and *P <* 0.01). Furthermore, interactions within diatoms and within dinoflagellates were eliminated. The networks were built using the “igraph” package (42). To assess bacterial interactions with dinoflagellates (Din-BN) and diatoms (Dia-BN), ASVs of phytoplankton and bacteria were chosen with a frequency of more than 10. The correlations between phytoplankton and bacteria were determined by Spearman’s correlation coefficients (|ρ| > 0.5 and *P <* 0.05). The networks were generated as described above and visualized using Gephi (43). In addition, network parameters, including modularity, average path length (APL), degree, closeness centrality, and betweenness centrality were assessed using Gephi. The bacterial dataset employed for this analysis was derived from our previous work (25).

## RESULTS

### Compositions of dinoflagellates and diatoms and their relationships with environmental factors

**Fig 1:**
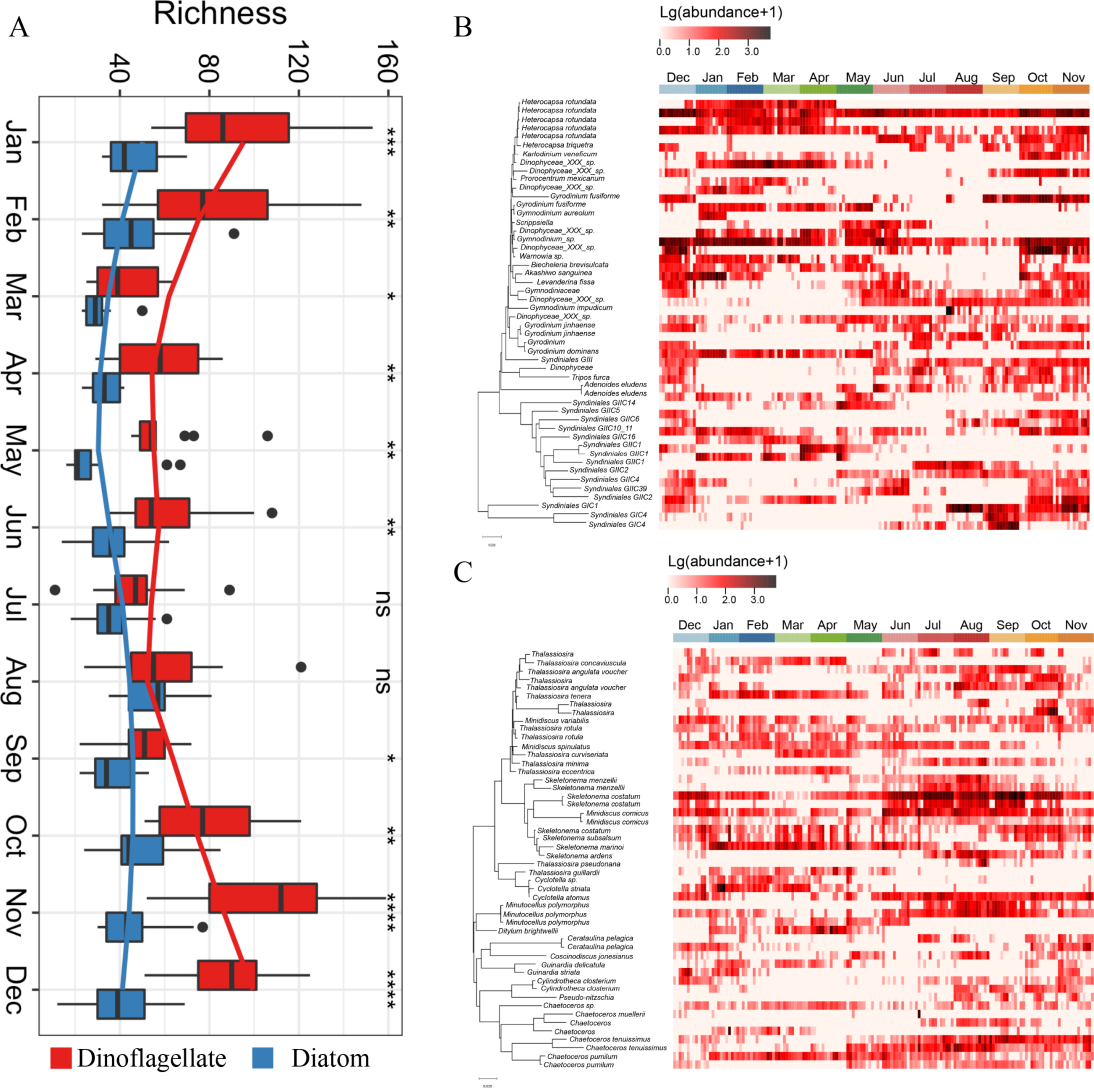
Temporal variations in the richness and relative abundance of dinoflagellates and diatoms across the whole year. (A) Richness. (B and C) Heatmap of relative abundance of dinoflagellates and diatoms, respectively. *P < 0.05; **P < 0.01; ***P < 0.001; ****P < 0.0001; ns: non-significant.

The average relative abundance of dinoflagellates and diatoms was 27.7% and 14.8%, respectively (Fig. S1). Furthermore, dinoflagellates exhibited significant seasonal variations in their richness, peaking in autumn and winter compared to spring and summer (Fig. 1A). Compared to dinoflagellates, diatoms had lower richness, with relatively minor seasonal fluctuations (Fig. 1A). Within top50 dinoflagellates, *Heterocapsa rotundata* and *Gymnodinium* sp. demonstrated the highest average relative abundance of 20.7% and 15.0%, respectively (Fig. 1B). For top50 diatoms, *Skeletonema costatum*, *Skeletonema marinoi*, and *Chaetoceros pumilum* dominated throughout the year. Specifically, *S. costatum* can maintain a relatively high abundance at high-temperature seasons (Fig. 1C).

The correlation between the abundance of dinoflagellates and temperature was relatively low (*R*^2^ = 0.027, *P* = 0.024), whereas the correlation between the abundance of diatoms and temperature was significantly positive (*R*^2^ = 0.201, *P* < 0.001; Fig. S2). However, dinoflagellates (*R*^2^ = 0.024, *P* = 0.03) are more influenced by the water NQI compared to diatoms (*R*^2^ = 0.004, *P* = 0.217; Fig. S2). From Spearman’s rank correlation, weak and negative correlations were observed between temperature and the total dinoflagellate community (ρ = −0.21, *P* < 0.05) as well as the top50 dinoflagellates (ρ = −0.20, *P* < 0.05; Fig. S3). In contrast, temperature was substantially positively associated with the total diatom community (ρ = 0.49, *P* < 0.05) and the top50 diatoms (ρ = 0.51, *P* < 0.05). Among the nutrient factors, nitrite had significant negative correlations with dinoflagellates but weak positive associations with diatoms (Fig. S3). Nitrate showed weak positive correlations with dinoflagellates but weak negative correlations with diatoms (Fig. S3).

### Niche breadth and seasonality of dinoflagellates and diatoms

**Fig 2:**
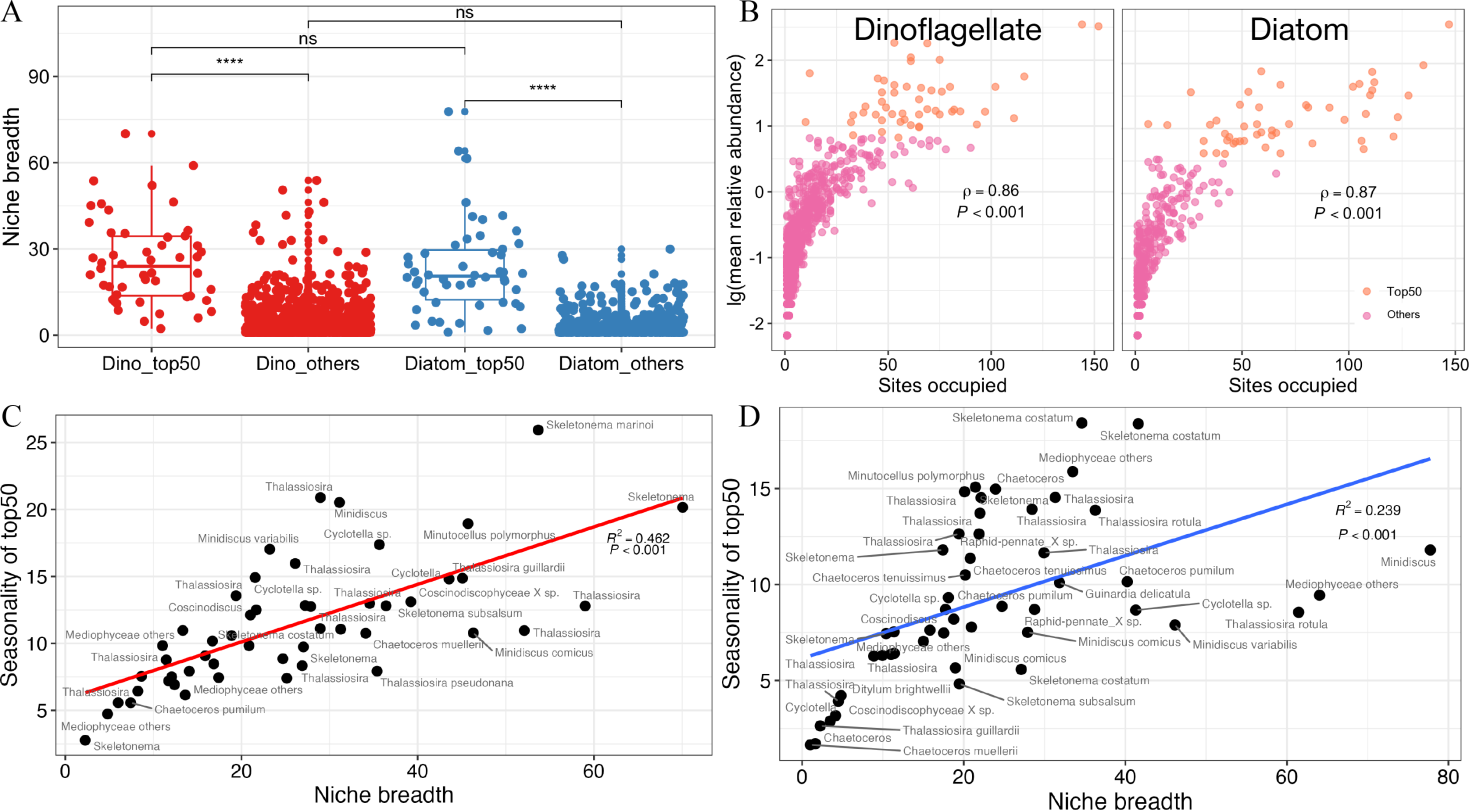
Niche breadth and sites occupancy of dinoflagellates and diatoms. (A) Niche breadth of the top50 ASVs and the remaining ASVs (others). (B) Sites occupancy of dinoflagellates (left) and diatoms (right). (C and D) Relationship between niche breadth and seasonality of top50 ASVs for dinoflagellates and diatoms, respectively.

The niche breadth for the top50 ASVs of dinoflagellates and diatoms did not exhibit any significant difference (Fig. 2A). However, the niche breadth of the top50 ASVs for both phytoplankton was substantially higher than that of the low-abundance taxa (*P* < 0.0001; Fig. 2A). Positive correlations were observed between sites occupancy and the average relative abundance of dinoflagellates (ρ = 0.86, *P* < 0.001) and diatoms (ρ = 0.87, *P* < 0.001; Fig. 2B). In addition, the site occupancy of the top50 ASVs for both phytoplankton was substantially higher than the low-abundance taxa (Fig. 2B). Niche breadth had a significant positive correlation with seasonality of the top50 of dinoflagellates (ρ = 0.462, *P* < 0.001) and diatoms (ρ = 0.239, *P* < 0.001; Fig. 2C and D).

### Factors mediating the variations in dinoflagellate and diatom communities

**Fig 3:**
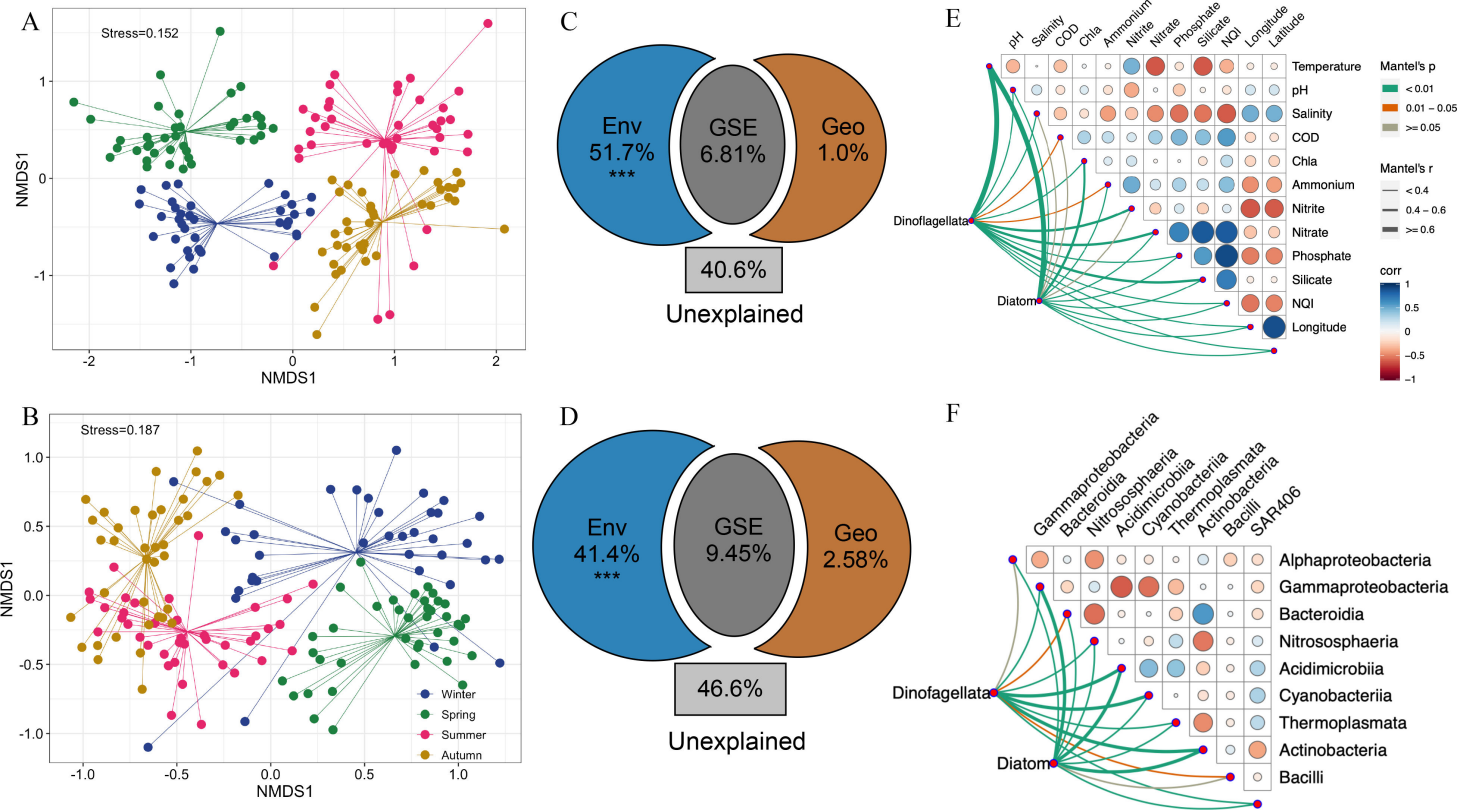
Variations of phytoplankton community structure. (A and B) NMDS plot depicting the community structure of dinoflagellates and diatoms, respectively. (C and D) VPA analyzing the explanation of environmental factors and geographic distance on the variation of dinoflagellate and diatom communities, respectively. (E) The relationship between environmental factors and phytoplankton community. (F) The relationship between bacterial community and phytoplankton community.

The NMDS analysis exhibited distinct patterns in the community structures of dinoflagellates and diatoms across different seasons (Fig. 3A and B), which was confirmed by the ADONIS analysis (Table S1). Approximately 51.7% of the variation in dinoflagellate communities and 41.4% of the variation in diatom communities were significantly explained by environmental factors (*P* < 0.001; Fig. 3C and D). The geographic factors had weak correlations with variations of phytoplankton communities. However, about 40.6% and 46.6% of the variations of dinoflagellate and diatom communities, respectively, remained unexplained. Mantel tests revealed that temperature is the most crucial abiotic factor (*P* < 0.01) influencing the year-round community structure variation of both phytoplankton (Fig. 3E).

Dinoflagellates were significantly correlated with temperature in autumn (ρ = 0.807, *P* < 0.001), followed by winter (ρ = 0.722, *P* < 0.001), summer (ρ = 0.531, *P* < 0.001), and spring (ρ = 0.503, *P* < 0.001; Table S2). Diatoms also had significant correlations with temperature in autumn (ρ = 0.512, *P* < 0.001), followed by winter (ρ = 0.455, *P* < 0.001), spring (ρ = 0.436, *P* < 0.001), and summer (ρ = 0.411, *P* < 0.001; Table S3). In terms of nutrients, the strongest correlations were observed between nitrate and the community variations, especially in summer (dinoflagellates: ρ = 0.586, *P* < 0.001; diatoms: ρ = 0.361, *P* < 0.001) and autumn (dinoflagellates: ρ = 0.660, *P* < 0.001; diatoms: ρ = 0.430, *P* < 0.001; Table S2 and S3). By analyzing the relationships among dinoflagellate and diatom communities and bacteria, noteworthy correlations were discovered with the compositions of Acidimicrobiia, Actinobacteria, and Cyanobacteriia (Fig. 3F).

### Assembly mechanisms of dinoflagellate and diatom communities

**Fig 4:**
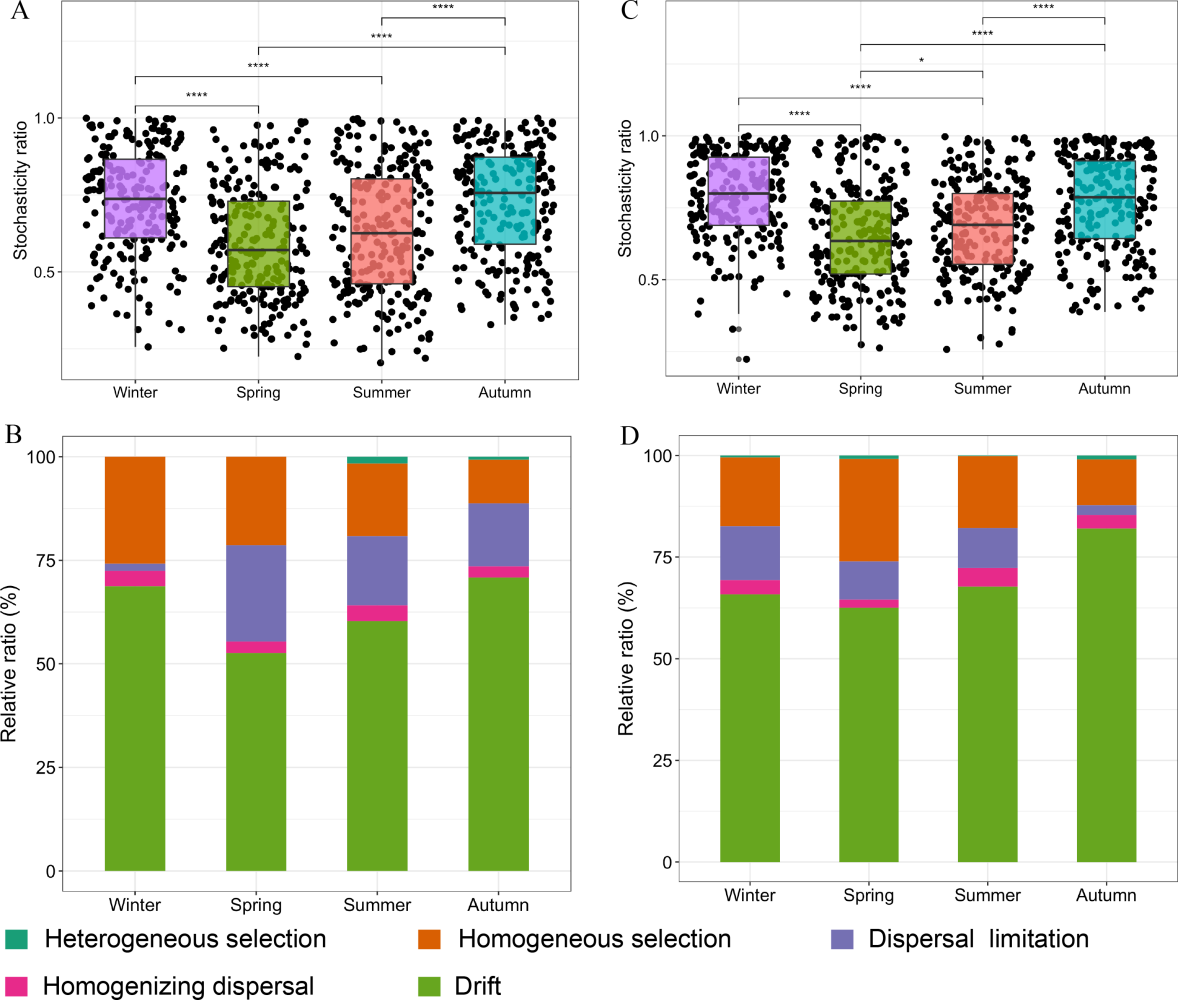
Assembly mechanisms of phytoplankton community. (A and C) Stochasticity ratio of dinoflagellates and diatoms, respectively. (B and D) Assembly mechanisms derived from βNTI and Raup-Crick index for dinoflagellates and diatoms, respectively. *P < 0.05; **P < 0.01; ***P < 0.001; ****P < 0.0001; ns: non-significant.

The ST values for dinoflagellate and diatom communities were greater than 0.5, indicating that both communities are predominantly driven by stochastic processes (Fig. 4A and C). Furthermore, the ST values were significantly higher in the autumn and winter compared to the spring and summer, suggesting stronger stochasticity in the autumn and winter. Moreover, ST values were markedly associated with the total (*R*^2^ = 0.545, *P* = 0.004) and low abundance (*R*^2^ = 0.558, *P* = 0.003) dinoflagellate communities, while the correlations with the top50 ASVs were not significant (*R*^2^ = 0.076, *P* = 0.197; Fig. S4). In contrast, the average abundance of diatoms had no significant correlation with the ST (Fig. S4). The community assembly processes for both phytoplankton were primarily dominated by drift, followed by homogeneous selection and dispersal limitation (Fig. 4B and D). Drift accounted for 52.7% and 62.6% of the community assembly processes for dinoflagellates and diatoms, respectively, in spring; 60.3% and 67.8% in summer; 70.8% and 82.0% in autumn; and 68.7% and 65.8% in winter. Except for winter, dispersal limitation had a greater impact on the community assembly process of dinoflagellates compared to diatoms.

### Co-occurrence networks between dinoflagellates and diatoms

**Fig 5:**
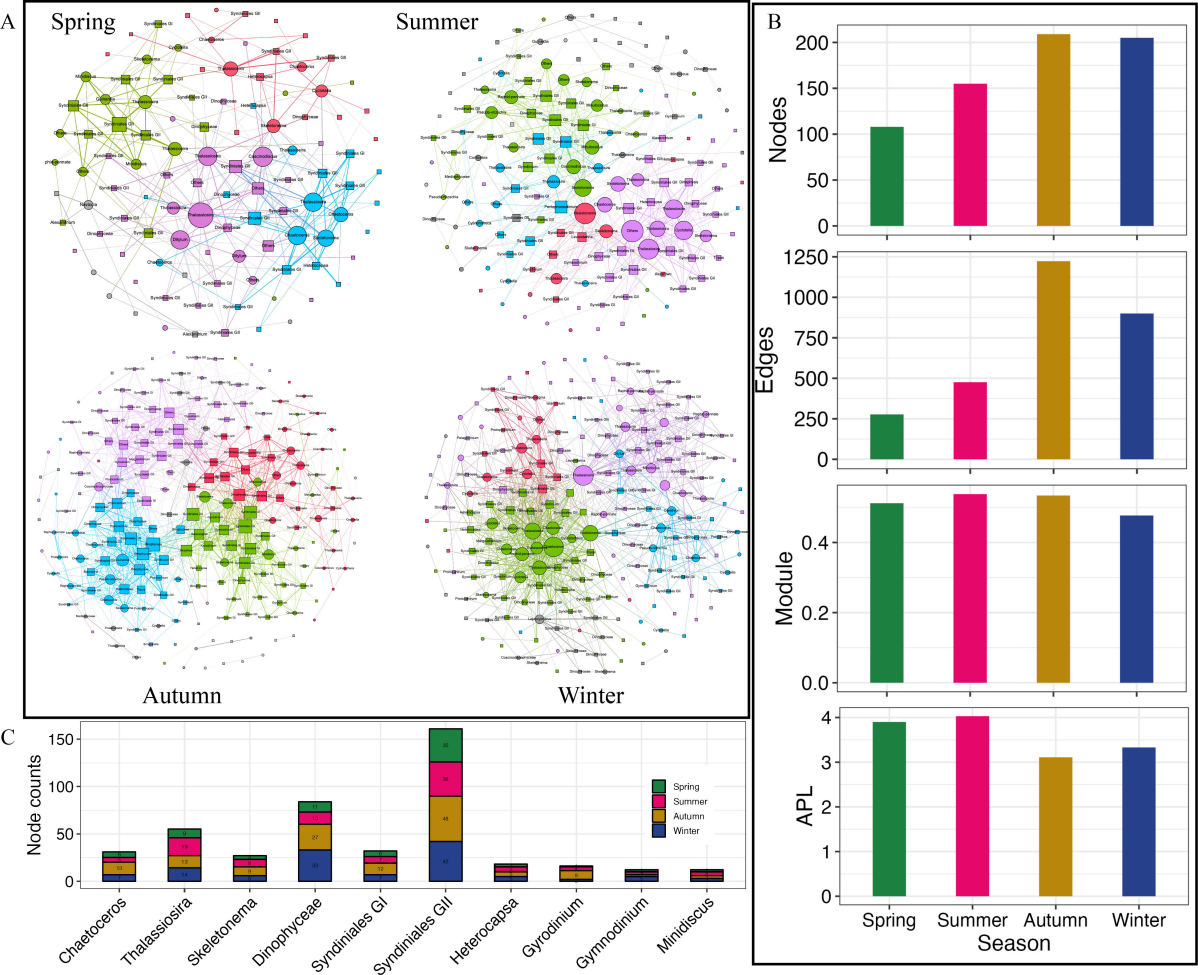
Co-occurrence patterns between dinoflagellates and diatoms. (A) Co-occurrence networks in the four seasons. (B) Network nodes, edges, modularity, and APL. (C) Compositions of networks.

The co-occurrence networks analysis revealed that the interactions between diatoms and dinoflagellates are the most complex in autumn, comprising a total of 209 nodes connected by 1,223 edges (Fig. 5A and B). This complexity is followed by winter, with 205 nodes connected by 900 edges, and summer, with 155 nodes connected by 476 edges. The interaction network in spring is the simplest, comprising 108 nodes connected by 277 edges (Fig. 5A and B). The modularity from spring to winter was 0.512, 0.538, 0.534, and 0.477 (Fig. 5B), respectively. The four networks comprised four major modules, with module I comprising the largest nodes, accounting for 31.5%, 28.4%, 24.4%, and 36.6% of the four networks from spring to winter, respectively (Table S4). The majority of connections were positive, with spring having the lowest positive ratio at 70.1% (Table S4).

Furthermore, in each network, dinoflagellates had more nodes than diatoms (Fig. 5A). Nodes of Syndiniales group II were the most abundant across all seasons, followed by *Dinophyceae*, *Thalassiosira*, *Chaetoceros*, and *Skeletonema* (Fig. 5C). Moreover, within each network module, *Thalassiosira*, *Skeletonema*, and *Chaetoceros* had the highest average degree in the spring, summer, and winter, indicating their critical roles in the networks. In addition, the network degree analysis also indicated that in spring, summer, and winter, the degree of diatoms was higher than that of dinoflagellates (Fig. S5). These findings suggested that although the node counts of diatoms in the networks were fewer than dinoflagellates, diatoms may play central roles in the network.

### Bacteria correlated to dinoflagellates and diatoms

**Fig 6:**
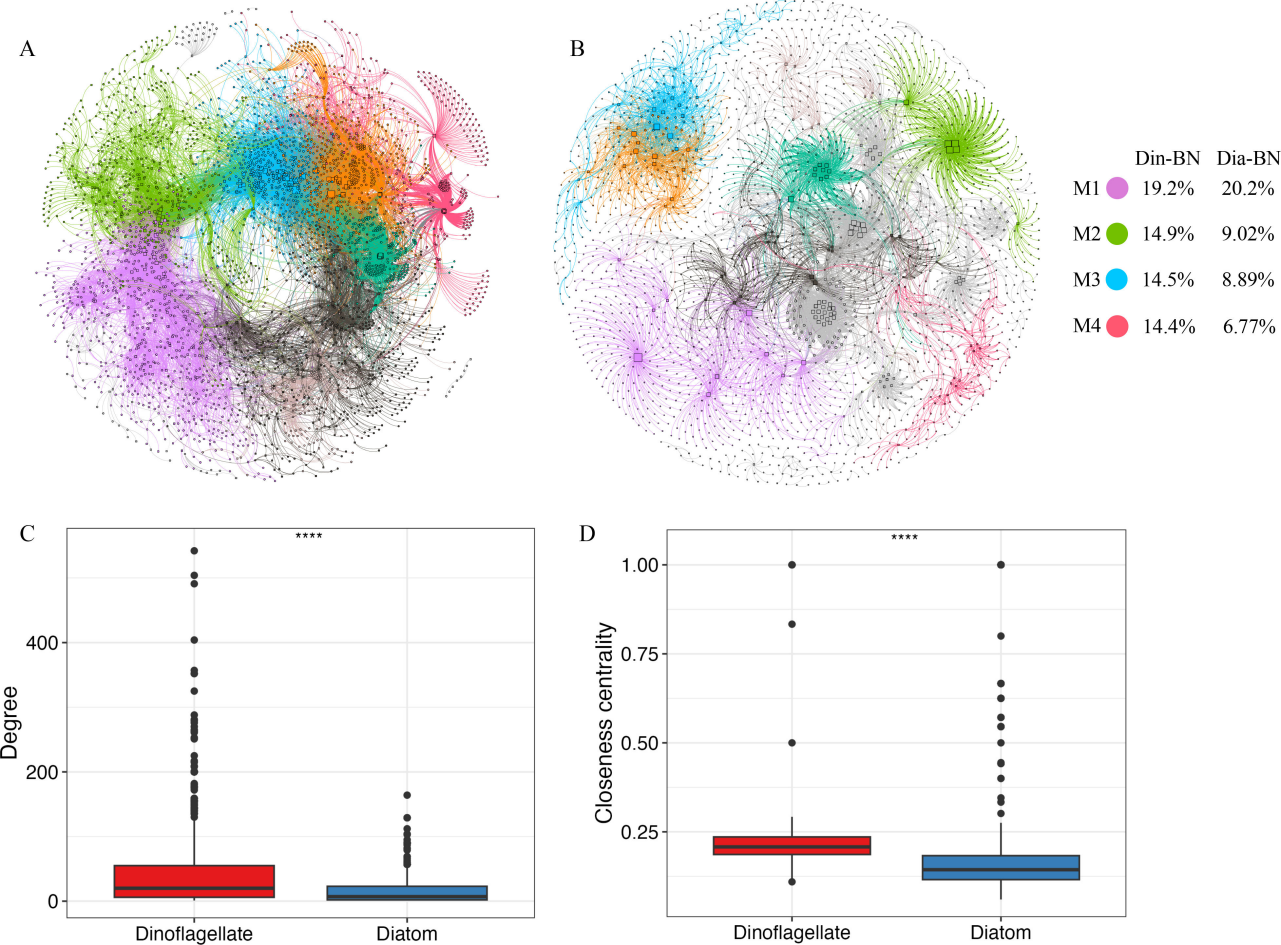
Co-occurrence patterns between dinoflagellates and bacteria (**A**), as well as diatoms and bacteria (**B**).(C) Network degree. (D) Closeness centrality. ****P < 0.0001.

Interactions of Din-BN exhibited significantly higher complexity than Dia-BN (Fig. 6A and B). The Din-BN interaction comprised 3,416 nodes with 26,071 edges, while Dia-BN had 2,261 nodes and only 7,903 edges (Fig. 6; Table S5). The average degree of the Din-BN was 15.3, significantly higher than that of Dia-BN (6.99). However, the modularity (0.744) and APL (4.81) of the Din-BN were lower than those of the Dia-BN (modularity: 0.866, APL: 7.56) (Table S5). Furthermore, network degree and closeness centrality of dinoflagellates were significantly (*P* < 0.001) higher than those of diatoms (Fig. 6C and D), highlighting that dinoflagellates have higher importance when interacting with bacteria.

*Rhodobacteraceae* and *Flavobacteriaceae* were the top two bacterial families with the highest interactions with dinoflagellates (122 and 121, respectively) and diatoms (84 and 83, respectively; Fig. S6), suggesting their close association with phytoplankton growth. In addition, there was a significant disparity in the types of bacterial families interacting with dinoflagellates and diatoms. Specifically, dinoflagellates primarily associated with family *Pirellulaceae* (44), Gemmatimonadota/BD2-11_terrestrial_group (45), Acidobacteriota/Subgroup_22 (45), *Woeseiaceae* (41), *Bdellovibrionaceae* (38), and *Halieaceae* (38) (Fig. S6). However, diatoms primarily associated with the family *Comamonadaceae* (33), *Halieaceae* (27), *Nitrosopumilaceae* (21), *Nitrosomonadaceae* (20), *Cyanobiaceae* (17), and SAR116_clade (17) (Fig. S6).

## DISCUSSION

The hydrological dynamics of XSB are intricately influenced by the combination of various factors, prominently the dilution effects caused by the freshwater influx from the Yangtze and Qiantang rivers, coupled with the influence of the Taiwan Warm Current (46). In coastal ecosystems, warming and eutrophication are the most critical environmental challenges, and variations in phytoplankton succession are mostly associated with temperature fluctuations and nutrient levels (17).

### Seasonal variations of community compositions of dinoflagellates and diatoms

It was observed that throughout the year, *Gymnodinium* sp. and *H. rotundata* were the most abundant dinoflagellates. *Gymnodinium* sp. exhibited significantly higher abundance during the spring and winter seasons compared to summer and autumn and has been reported to cause blooms in XSB (47). Furthermore, it can grow in a temperature range of 10°C–32°C (45), validating its enhanced abundance in XSB throughout the year. *Heterocapsa* sp. usually induces red tides in eutrophic bays and commonly thrives under low-temperature conditions (48). Syndiniales are usually categorized into five groups (49), and groups I and II were predominant in XSB; however, they had distinct seasonal patterns, with group I dominating in summer and autumn, while group II was predominantly in winter and spring. Diatoms play pivotal roles as primary food sources for both heterotrophic plankton and aquaculture organisms (50). In nutrient-rich environments, their remarkable reproductive activity enables rapid proliferation, often triggering harmful algal blooms along coastal regions (51). *S. costatum*, which has eurythermal and euryhaline characteristics, exhibits a broad distribution, especially in tropical and subtropical eutrophic waters (52). *Chaetoceros* is also a globally distributed alga that is frequently abundant in coastal harbors throughout China (53). Its growth is significantly facilitated by high temperatures during summer (54).

Ocean warming and increased anthropogenic nutrient influx are two major stressors associated with global changes in coastal waters (55). Our results found that temperature emerges as a predominant factor that modulates community compositions, which is consistent with evidence suggesting that global temperatures significantly influence microbial community compositions (5, 56). However, temperature may have different effects on dinoflagellates and diatoms. It was reported that diatoms have a wider adaptive temperature range than dinoflagellates (57). In addition, diatoms usually display higher nitrate utilization efficiency than dinoflagellates because diatoms have larger internal nitrate pools, highly active nitrate reductase, and a higher proportion of nitrate transporters on their cell membranes (23, 58). Furthermore, both these phytoplankton respond differently to ammonium concentrations (58, 59). The overall variations observed in the community compositions of diatoms and dinoflagellates during the four seasons are attributed to the algal physiological traits and environmental conditions. However, more than 40% of these variations cannot be explained by environmental factors, suggesting that other unmeasured abiotic factors or microbial interactions could significantly alter community structure. Therefore, additional study on other environmental factors is necessary for a comprehensive understanding of the community variation.

### The community assembly mechanisms of dinoflagellates and diatoms

Compared to less abundant species, the top50 ASVs of both dinoflagellates and diatoms exhibited greater niche breadth and higher site occupancy, typically identifying them as generalists. Due to their broad ecological niche and metabolic adaptability, generalists can utilize diverse resources for propagation (60). The top50 species usually exhibited strong seasonality, suggesting that they can adeptly adjust to environmental variations, resulting in increased regional presence within the meta-community due to their superior growth rates (61). Moreover, species with high abundance may possess a greater tolerance to environmental variations, making them more resilient to changing conditions (62). Altogether, these mechanisms significantly increase the population size and geographical spread of abundant species. In contrast, low-abundance species occupy fewer sites in the XSB and are typically identified as specialists. It has been observed that specialists exhibit (i) limited dispersal capabilities due to their lower abundances, (ii) narrow niche breadth and low competitiveness, as well as (iii) enhanced susceptibility to environmental selection pressures as they are highly adapted to their optimal habitat (61, 62). Overall, these findings indicated a reduced occurrence of low-abundance species across the sampling sites in the XSB.

Both deterministic and stochastic processes are found in the assembly of dinoflagellates and diatoms, but stochastic processes were dominant. This might result from the overall eutrophic conditions in the XSB, which facilitates the growth of phytoplankton with relatively low-competitive pressure. Furthermore, drift was observed as a primary force governing the assembly of these two phytoplankton, with a particularly pronounced effect on diatom communities. Drift has been found to play a significant role in shaping the microbial eukaryotic community in surface seawater of tropical and subtropical regions (11). Random migration, reproduction, birth, and death are the predominant factors responsible for drift, which results in stochastic fluctuations in microbial relative abundance (10). Therefore, during seasonal phytoplankton blooms in the XSB (63), changes in relative abundances of diatoms and dinoflagellates may amplify the roles of drift. Diatoms and dinoflagellates have adapted to the long-term eutrophic conditions in the XSB, which could induce weak environment selection. Thus, at small geographic scales and high eutrophic conditions, such as those observed in the XSB, drift has an essential impact on the phytoplankton community.

### Co-occurrence patterns variation between dinoflagellates and diatoms

Network analysis is a valuable tool for delineating the intrinsic biotic interactions between microbes (64). Here, a variety of distinct co-occurrence patterns were revealed between dinoflagellates and diatoms. During autumn and winter, there were growing interactions between diatoms and dinoflagellates, whereas an opposite trend was observed in spring and summer. The correlations between dinoflagellates and diatoms were significantly positive, which could be explained by niche overlap or co-colonization (65). Therefore, the low positive interactions in spring may suggest higher competition or cross-feeding among phytoplankton than during other seasons.

Interestingly, the most robust positive correlations were observed between Syndiniales and other algae. These parasites can transfer more than 50% of host biomass from the traditional food web to the microbial loop (66). Furthermore, previous studies have indicated that Syndiniales has a positive correlation with Chl *a* levels and was potentially associated with enhanced favorable production conditions or photosynthetic host biomass (67). This correlation may help explain its dominant role in the network. Moreover, Syndiniales are generalist and opportunistic parasites that can infect various marine hosts across different trophic levels (29). Small-size dinoflagellates, including *Gymnodinium* and *Heterocapsa* species, are potential hosts for Syndiniales (29). Dinoflagellates can survive in harsh environments because of the presence of cysts and typically have relatively low adaptation to environmental changes (68). Here, the average relative abundance of mixotrophic dinoflagellates, including species like *Gymnodinium* and *Heterocapsa*, was remarkably high in the XSB. When nutrients are sufficient, these dinoflagellates predominantly function as phototrophs; however, during less favorable environments, they resort to consuming prey (69). As a dominant diatom species in the XSB, *S. costatum* also served as a potential host for Syndiniales and as a food source for mixotrophic or heterotrophic dinoflagellates (29, 44). Furthermore, based on nutrient levels, diatoms and dinoflagellates can intensively interact with each other because diatoms typically show a positive correlation with nitrate and a negative association with ammonium, while the opposite scenario is usually observed in dinoflagellates (23). Therefore, it was speculated that algal interactions may be influenced not only by trophic type but also by nutrient availability.

The Din-BN interaction was more complex than the Dia-BN interaction. In terms of growth rate (70) and types of dissolved organic matter (DOM) release (71), the ecological traits of diatoms and dinoflagellates are largely different. Therefore, diatoms and dinoflagellates may have different co-occurrence patterns with bacterial communities. The consistently high year-round abundances of dinoflagellates contributed more to the overall biomass compared to diatoms, resulting in more intricate and complex Din-BN interactions. Among the bacteria ASVs, *Flavobacteriaceae* and *Rhodobacteraceae* had the largest connections with dinoflagellates and diatoms, suggesting their essential roles in the XSB. Flavobacteria can degrade high-molecular-weight DOM (72), and they are recognized as a significant component of the microbial loop in coastal phytoplankton blooms (20, 72). In addition to *Flavobacteriaceae*, *Rhodobacteraceae* can degrade various low-molecular-weight DOM from phytoplankton (72). During phytoplankton bloom, *Rhodobacteraceae* usually acts as a “pioneer” bacterium, fulfilling two crucial ecological roles within the phycosphere. First, they become the most abundant species in the natural environment by effectively competing and thriving under low-nutrient conditions (73). Second, they provide sulfur compounds that promote algal growth, thereby further contributing to the phytoplankton dynamics (74). Therefore, the associated bacteria, such as *Flavobacteriaceae* and *Rhodobacteraceae*, typically engage in mutualistic relationships with phytoplankton.

### Conclusions

The XSB has experienced long-standing eutrophication, marked by yearly outbreaks of dinoflagellate and diatom blooms. This study comprehensively investigated the composition and succession of these phytoplankton in the XSB, providing a more diverse species composition compared to that obtained from traditional microscopic examinations (17). The prolonged eutrophic conditions in the XSB can increase the stochastic processes in the community assembly. Interactions between dinoflagellates and diatoms exhibited notable seasonal variations. *Flavobacteriaceae* and *Rhodobacteraceae* are the bacterial taxa most closely associated with dinoflagellates and diatoms, indicating their crucial role in the consumption of algal organic matter. Overall, this study integrated data on community compositions, assembly, and interactions of diatoms and dinoflagellates, providing evidence that increases the understanding of the impact of long-term eutrophication on phytoplankton blooms in coastal bays. However, we agree that the 18S rRNA gene copy numbers vary among dinoflagellates and diatoms, potentially leading to an overestimation of their relative abundance (75). Although this limitation is unavoidable when using amplicon sequencing, employing a species-specific 18S rRNA gene copy number database may help mitigate this bias.

## SUPPLEMENTAL MATERIAL

Supplemental File msphere.00366-24-s0001.docx Tables S1 to S5; Fig. S1 to S6.

ASM does not own the copyrights to Supplemental Material that may be linked to, or accessed through, an article. The authors have granted ASM a non-exclusive, world-wide license to publish the Supplemental Material files. Please contact the corresponding author directly for reuse.
