# Supplementary material for: Interaction patterns and assembly mechanisms of dinoflagellates and diatoms in a coastal bay suffering from long-term eutrophication
Source: mSphere. 2024 Jun 28;9(7):e00366-24. doi: 10.1128/msphere.00366-24 (PMC11288026; doi:10.1128/msphere.00366-24)
Supplement: Supplemental File — Tables S1 to S5; Fig. S1 to S6. [file msphere.00366-24-s0001.docx]

**Interaction patterns and assembly mechanisms of dinoflagellates and diatoms in a coastal bay suffering from long-term eutrophication**

Wenfei Miao ^a,1^, Shuqi Wang ^a,1^, Tenghui Lin ^a^, Yi Yan ^a^, Zhen Bao ^a^, Demin Zhang ^a,b,c^, Zhibing Jiang ^d^, Huajun Zhang ^a,b,c*^

^a^ School of Marine Sciences, Ningbo University, Ningbo, 315211, China

^b^ State Key Laboratory for Managing Biotic and Chemical Threats to the Quality and Safety of Agro-products, Institute of Plant Virology, Ningbo University, Ningbo 315211, China

^c^ Key Laboratory of Applied Marine Biotechnology of Department of Education, Ningbo University, Ningbo, 315211, China

^d^ State Key Laboratory of Satellite Ocean Environment Dynamics, Second Institute of Oceanography, Ministry of Natural Resources, Hangzhou, 310012, China

^1^ These authors contributed equally to this work.

* For correspondence, E-mail: zhanghuajun@nbu.edu.cn (Huajun Zhang)

**Table S1** Permutational multivariate analysis of variance (ADONIS) showing differences in phytoplankton composition between seasons

|  | **Dinoflagellate** | | **Diatom** | |
| --- | --- | --- | --- | --- |
|  | R^2^ | *P* | R^2^ | *P* |
| Winter vs Spring | 0.145 | 0.001 | 0.089 | 0.001 |
| Winter vs Summer | 0.234 | 0.001 | 0.226 | 0.001 |
| Winter vs Autumn | 0.238 | 0.001 | 0.152 | 0.001 |
| Spring vs Summer | 0.175 | 0.001 | 0.323 | 0.001 |
| Spring vs Autumn | 0.246 | 0.001 | 0.245 | 0.001 |
| Summer vs Autumn | 0.143 | 0.001 | 0.108 | 0.001 |

**Table S2** Mantel tests showing the correlations between environmental factors (Euclidean distance) and dinoflagellate β-diversity (Bray–Curtis distance) with 999 permutations

|  | Spring | | Summer | | Autumn | | Winter | |
| --- | --- | --- | --- | --- | --- | --- | --- | --- |
|  | 𝜌 | *P* | 𝜌 | *P* | 𝜌 | *P* | 𝜌 | *P* |
| Temperature | 0.503 | 0.001 | 0.531 | 0.001 | 0.807 | 0.001 | 0.722 | 0.001 |
| Salinity | 0.237 | 0.003 | 0.254 | 0.001 | 0.456 | 0.001 | 0.212 | 0.001 |
| pH | 0.292 | 0.001 | 0.434 | 0.002 | 0.337 | 0.001 | 0.092 | 0.13 |
| COD | 0.368 | 0.001 | 0.042 | 0.242 | 0.205 | 0.001 | 0.068 | 0.151 |
| Chl a | -0.016 | 0.554 | 0.237 | 0.001 | 0.034 | 0.247 | 0.213 | 0.007 |
| Ammonium | 0.036 | 0.311 | 0.179 | 0.018 | 0.176 | 0.019 | 0.183 | 0.009 |
| Nitrite | 0.259 | 0.001 | 0.188 | 0.004 | 0.431 | 0.001 | 0.248 | 0.002 |
| Nitrate | 0.145 | 0.032 | 0.586 | 0.001 | 0.660 | 0.001 | 0.234 | 0.002 |
| Phosphate | 0.080 | 0.146 | 0.236 | 0.003 | 0.422 | 0.001 | 0.441 | 0.001 |
| Silicate | 0.247 | 0.001 | 0.352 | 0.001 | 0.676 | 0.006 | 0.418 | 0.001 |

**Table S3** Mantel tests showing the correlations between environmental factors (Euclidean distance) and diatom β-diversity (Bray–Curtis distance) with 999 permutations

|  | Spring | | Summer | | Autumn | | Winter | |
| --- | --- | --- | --- | --- | --- | --- | --- | --- |
|  | 𝜌 | *P* | 𝜌 | *P* | 𝜌 | *P* | 𝜌 | *P* |
| Temperature | 0.436 | 0.001 | 0.411 | 0.001 | 0.512 | 0.001 | 0.455 | 0.001 |
| Salinity | 0.110 | 0.053 | 0.273 | 0.001 | 0.276 | 0.002 | 0.162 | 0.025 |
| pH | 0.213 | 0.002 | 0.253 | 0.002 | 0.262 | 0.001 | 0.181 | 0.044 |
| COD | 0.341 | 0.001 | 0.087 | 0.118 | 0.120 | 0.024 | 0.006 | 0.446 |
| Chl a | 0.238 | 0.002 | 0.166 | 0.016 | 0.146 | 0.022 | 0.400 | 0.001 |
| Ammonium | 0.052 | 0.207 | 0.093 | 0.171 | 0.187 | 0.009 | 0.188 | 0.019 |
| Nitrite | 0.204 | 0.001 | 0.322 | 0.002 | 0.157 | 0.008 | 0.157 | 0.021 |
| Nitrate | 0.193 | 0.003 | 0.361 | 0.001 | 0.430 | 0.001 | 0.077 | 0.186 |
| Phosphate | 0.068 | 0.162 | 0.355 | 0.001 | 0.378 | 0.001 | 0.227 | 0.006 |
| Silicate | 0.220 | 0.003 | 0.184 | 0.007 | 0.377 | 0.001 | 0.339 | 0.001 |

**Table S4** Modules and connections of networks between dinoflagellates and diatoms in each season

|  | Spring | Summer | Autumn | Winter |
| --- | --- | --- | --- | --- |
| **M1** | 31.5% | 28.4% | 24.4% | 36.6% |
| **M2** | 22.2% | 24.5% | 21.1% | 25.4% |
| **M3** | 19.4% | 16.1% | 20.1% | 16.6% |
| **M4** | 19.4% | 10.3% | 19.1% | 11.7% |
| **Positive** | 195 | 394 | 1076 | 739 |
| **Negative** | 83 | 84 | 149 | 163 |

**Table S5** Topological features of the co-occurrence patterns between phytoplankton and bacteria

|  | **Nodes** | **Edges** | **Module** | **APL** | **Average degree** |
| --- | --- | --- | --- | --- | --- |
| **Dinoflagellate** | 3416 | 26071 | 0.744 | 4.81 | 15.3 |
| **Diatom** | 2261 | 7903 | 0.866 | 7.56 | 6.99 |

**Figures**


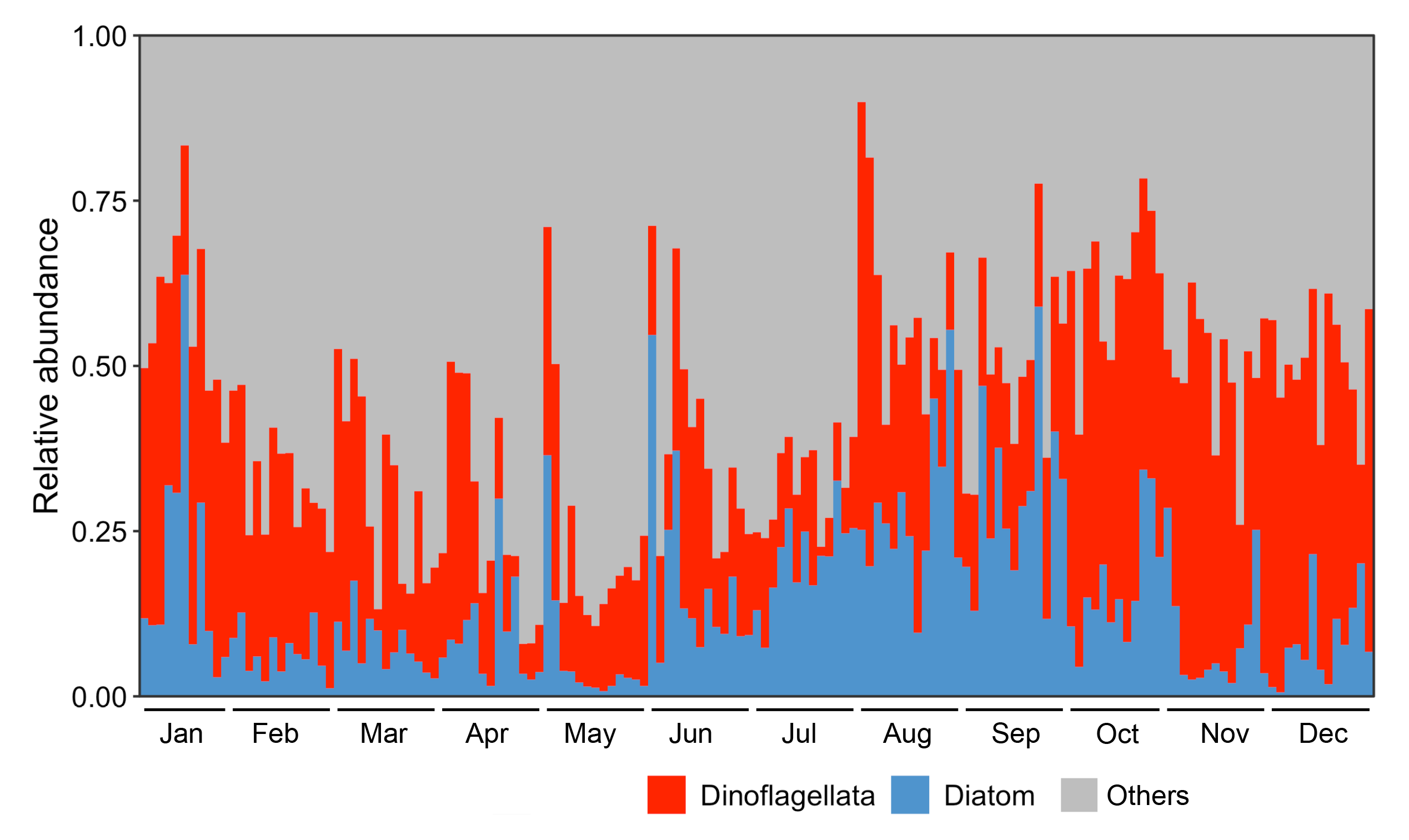


**Fig. S1** Relative abundances of dinoflagellates and diatoms throughout the whole year.


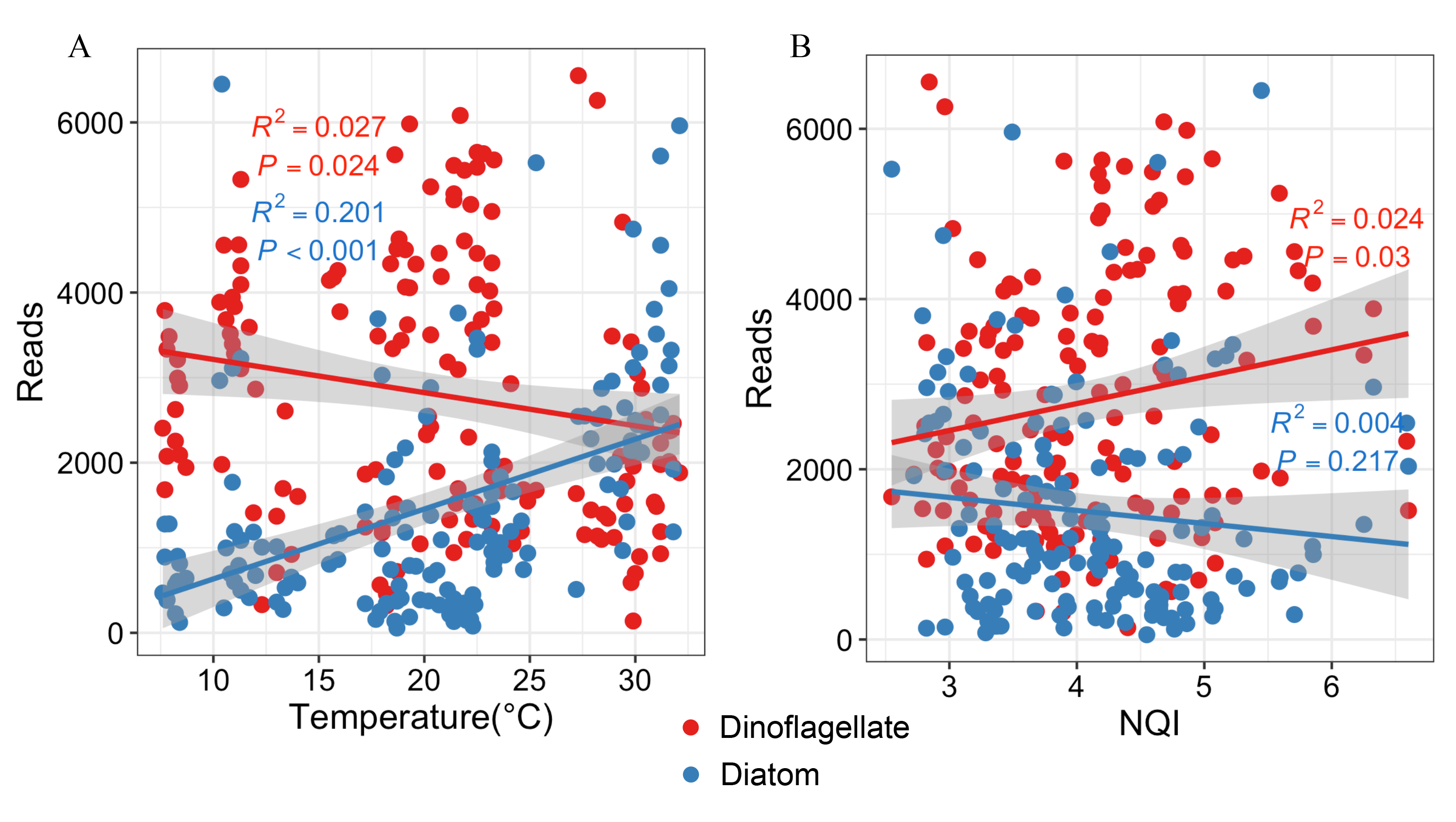


**Fig. S2** Relationship between phytoplankton and temperature (A), as well as phytoplankton and NQI (B).


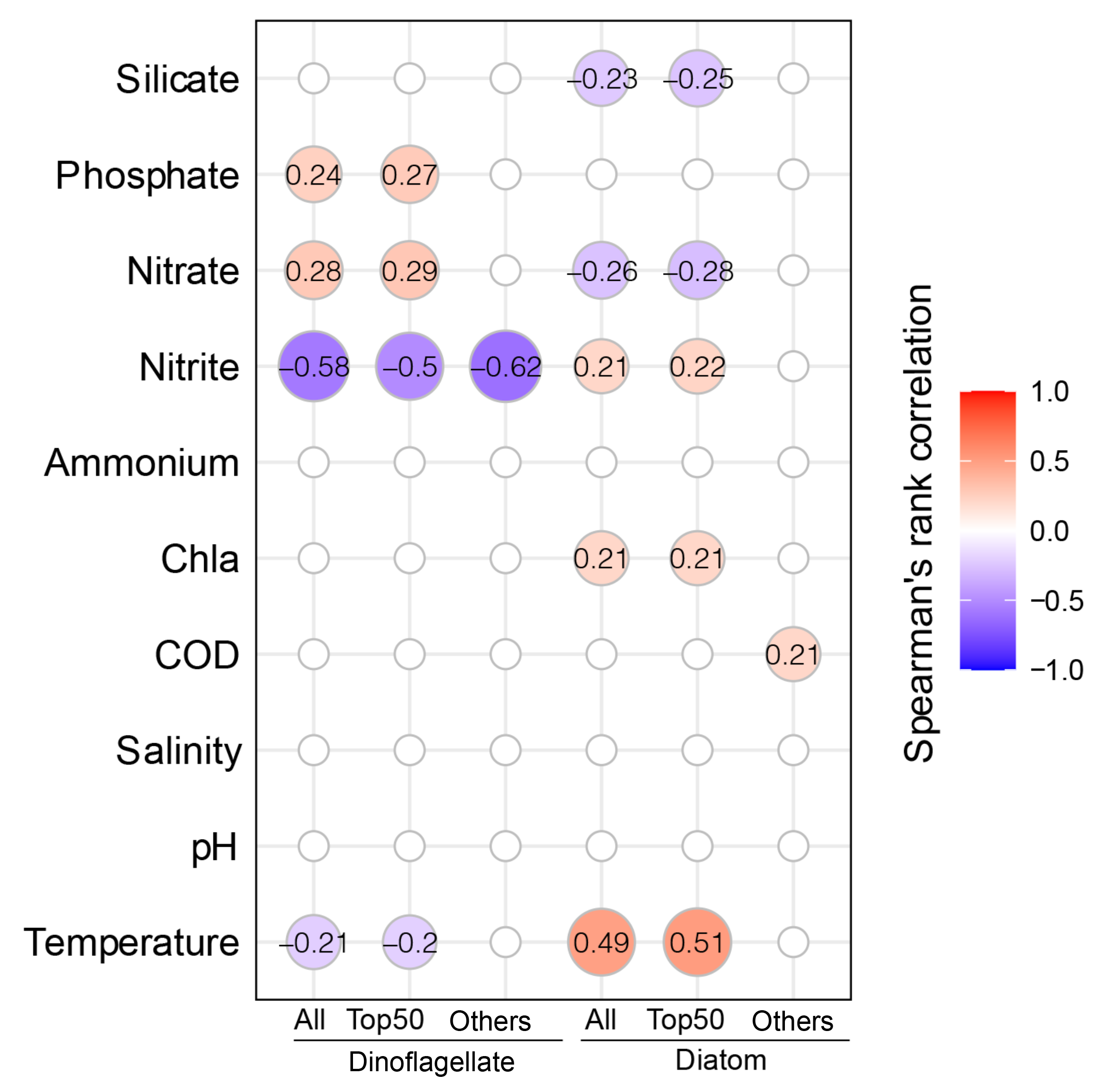


**Fig. S3** Relationship between environmental factors and phytoplankton based on the spearman’s rank correlation. Only significant correlations were displayed. All: total phytoplankton community; Top50: the top50 ASVs; Others: ASVs excluding the top50 ASVs.


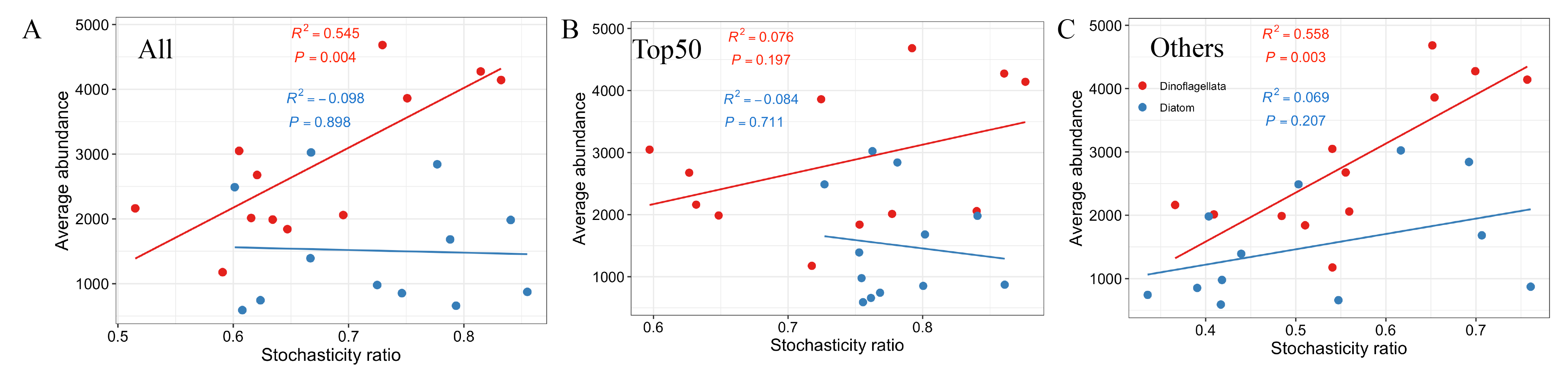


**Fig. S4** Relationship between stochasticity ratio and average relative abundance of dinoflagellates and diatoms. A: total phytoplankton community; B: the top50 ASVs; C: ASVs excluding the top50 ASVs.


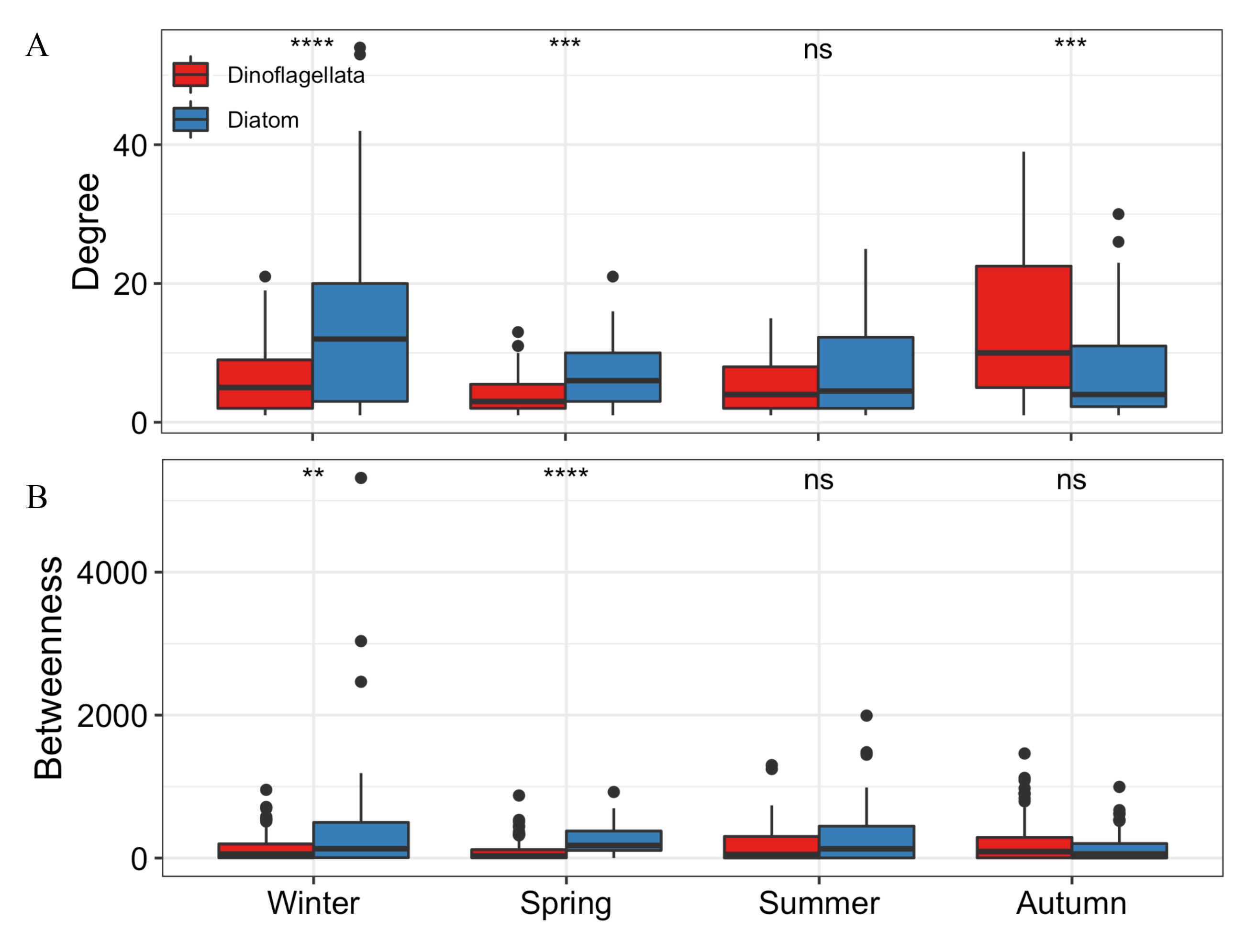


**Fig. S5** Network parameters of interactions between dinoflagellates and diatoms.

** *P* < 0.01; *** *P* < 0.001; **** *P* < 0.0001. ns: non-significant.


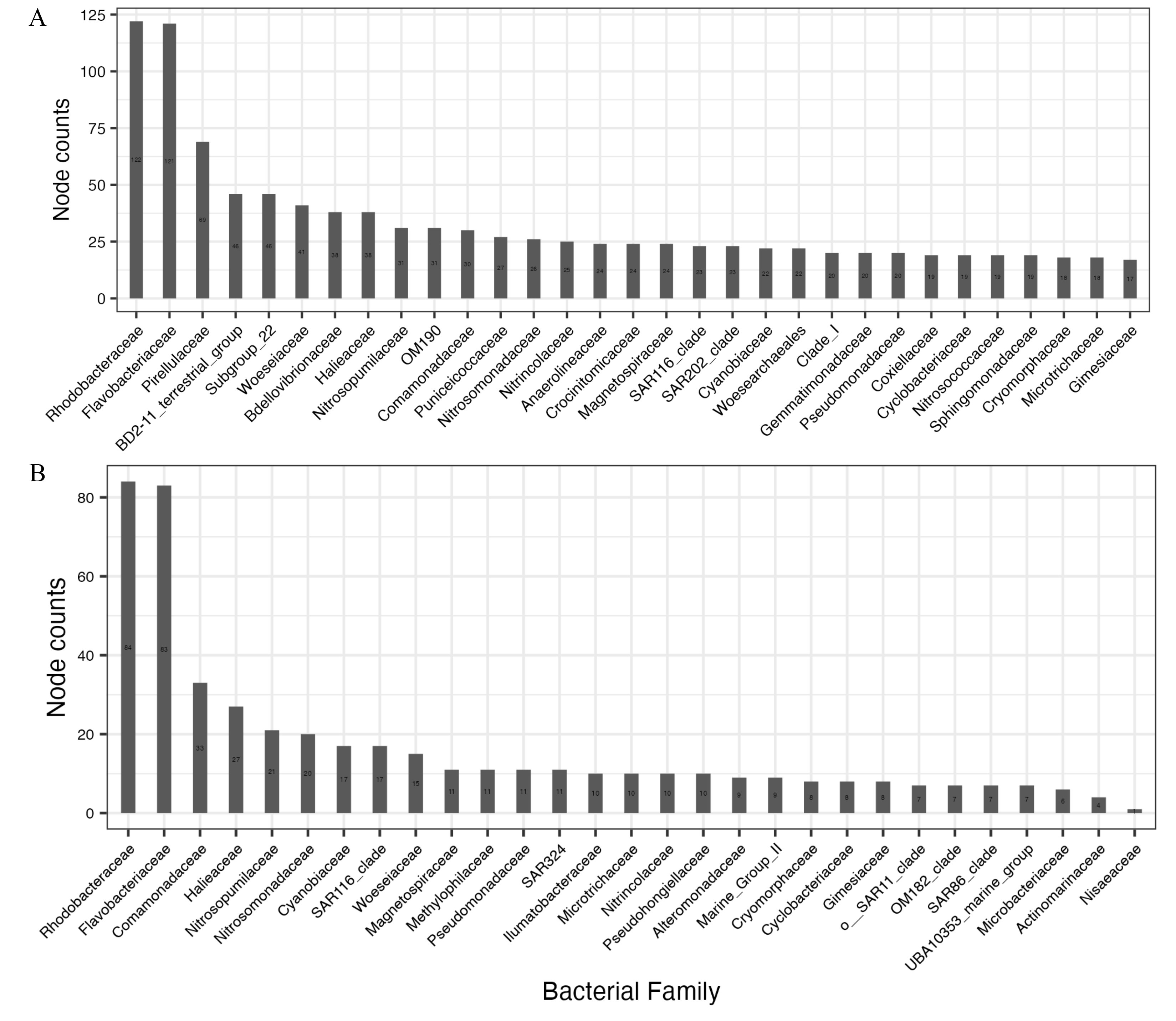


**Fig. S6** Bacteria associated with dinoflagellates (A) and diatoms (B).
